# Aqua­(cyanido-κ*C*){6,6′-dimeth­oxy-2,2′-[*o*-phenyl­enebis(nitrilo­methanylyl­idene)]diphenolato-κ^4^ *O* ^1^,*N*,*N*′,*O* ^1′^}cobalt(III) acetonitrile monosolvate

**DOI:** 10.1107/S1600536811029461

**Published:** 2011-07-30

**Authors:** Yang Lin, Guang-Ming Li, Peng Chen, Peng-Fei Yan, Guang-Feng Hou

**Affiliations:** a Key Laboratory of Functional Inorganic Materials Chemistry (MOE), School of Chemistry and Materials Science, Heilongjiang University, Harbin 150080, People’s Republic of China

## Abstract

In the title complex, [Co(C_22_H_18_N_2_O_4_)(CN)(H_2_O)]·CH_3_CN, the Co^III^ ion is six-coordinated in a distorted octa­hedral environment defined by two N atoms and two O atoms from a salen ligand in the equatorial plane and one O atom from a water mol­ecule and one C atom from a cyanide group at the axial positions. O—H⋯O hydrogen bonds connect adjacent complex mol­ecules into dimers. C—H⋯N hydrogen bonds and π–π inter­actions between the benzene rings [centroid–centroid distances = 3.700 (2) and 3.845 (2) Å] are also present.

## Related literature

For the synthesis of the ligand, see: Costes *et al.* (2000 ▶). For related transition-metal complexes, see: Przychodzeń *et al.* (2005 ▶). For bond-valence calculations, see: Spek (2009 ▶).
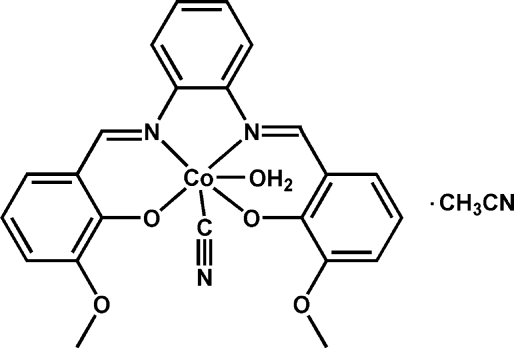


## Experimental

### 

#### Crystal data

[Co(C_22_H_18_N_2_O_4_)(CN)(H_2_O)]·C_2_H_3_N
*M* *_r_* = 518.40
Monoclinic, 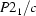

*a* = 10.829 (2) Å
*b* = 13.209 (3) Å
*c* = 18.906 (6) Å
β = 118.30 (2)°
*V* = 2381.1 (10) Å^3^
*Z* = 4
Mo *K*α radiation
μ = 0.77 mm^−1^
*T* = 293 K
0.34 × 0.31 × 0.29 mm

#### Data collection

Rigaku R-AXIS RAPID diffractometer
Absorption correction: multi-scan (*ABSCOR*; Higashi, 1995 ▶) *T*_min_ = 0.780, *T*_max_ = 0.811
22543 measured reflections
5426 independent reflections
4439 reflections with *I* > 2σ(*I*)
*R*_int_ = 0.034

#### Refinement

*R*[*F* ^2^ > 2σ(*F* ^2^)] = 0.034
*wR*(*F* ^2^) = 0.096
*S* = 1.08
5426 reflections
319 parameters
H-atom parameters constrained
Δρ_max_ = 0.36 e Å^−3^
Δρ_min_ = −0.35 e Å^−3^

### 

Data collection: *RAPID-AUTO* (Rigaku, 1998 ▶); cell refinement: *RAPID-AUTO*; data reduction: *CrystalStructure* (Rigaku/MSC, 2002 ▶); program(s) used to solve structure: *SHELXS97* (Sheldrick, 2008 ▶); program(s) used to refine structure: *SHELXL97* (Sheldrick, 2008 ▶); molecular graphics: *DIAMOND* (Brandenburg, 1999 ▶); software used to prepare material for publication: *SHELXTL* (Sheldrick, 2008 ▶).

## Supplementary Material

Crystal structure: contains datablock(s) I, global. DOI: 10.1107/S1600536811029461/hy2451sup1.cif

Structure factors: contains datablock(s) I. DOI: 10.1107/S1600536811029461/hy2451Isup2.hkl

Additional supplementary materials:  crystallographic information; 3D view; checkCIF report

## Figures and Tables

**Table 1:** Selected bond lengths (Å)

Co1—C23	1.869 (2)
Co1—N1	1.8944 (15)
Co1—N2	1.8972 (16)
Co1—O1	1.8948 (13)
Co1—O2	1.8998 (14)
Co1—O5	2.0194 (14)

**Table 2:** Hydrogen-bond geometry (Å, °)

*D*—H⋯*A*	*D*—H	H⋯*A*	*D*⋯*A*	*D*—H⋯*A*
O5—H51⋯O3^i^	0.85	2.33	2.922 (2)	127
O5—H51⋯O1^i^	0.85	2.00	2.799 (2)	156
O5—H52⋯O2^i^	0.85	2.24	2.813 (2)	124
O5—H52⋯O4^i^	0.85	2.10	2.902 (2)	158
C10—H10⋯N4^ii^	0.93	2.61	3.433 (3)	148
C15—H15⋯N3	0.93	2.56	3.440 (3)	159

Symmetry codes: (i) 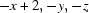; (ii) 
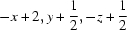
.

**Fig. 1.**
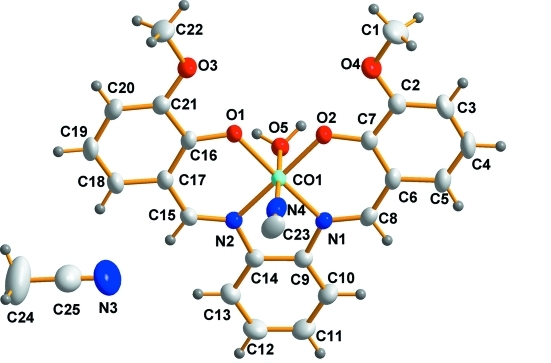
The molecular structure of the title compound. Displacement ellipsoids are drawn at the 50% probability level.

**Fig. 2.**
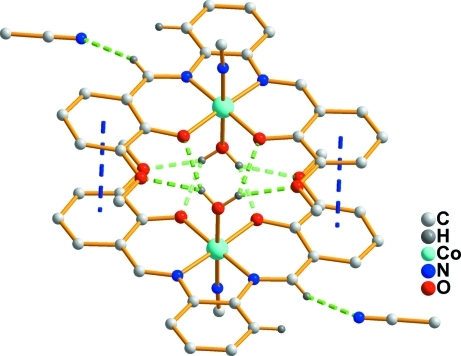
A view of the hydrogen-bonded dimer, showing hydrogen bonds (green dashed lines) and π–π interactions (blue dashed lines).

## supplementary crystallographic information

### Comment

Transition metal complexes with spectroscopic and magnetic properties are currently of considerable interest. As a continuing work for the studies of salen ligands (Costes *et al.*, 2000) and transition metal complexes (Przychodzeń *et al.*, 2005), we present here the synthesis and crystal structure of the title compound.

The bond valence calculation (Spek, 2009) indicated that the Co atom is in a 3+ state, which can be produced by Li(TCNQ) oxidating Co(II) atom [TCNQ = 2,2'-(2,5-cyclohexadiene-1,4-diylidene)bis(propanedinitrile)]. Meanwhile, TCNQ decomposed to produce cyanide group. In the title complex, the Co^III^ ion is six-coordinated in a distorted octahedral environment defined by two imino N atoms and two phenolate O atoms from the salen type ligand, one O atom from a water molecule and one C atom from a cyanide group (Fig. 1, Table 1). O—H···O hydrogen bonds connect two adjacent complex molecules into a dimer (Fig. 2, Table 2). C—H···N hydrogen bonds and π–π interactions between the benzene rings [centroid–centroid distance = 3.700 (2) and 3.845 (2) Å] are present.

### Experimental

A solution of Co*L* (0.078 g, 0.1 mmol) [*L* = *N*,*N*'-bis(3-methoxy-2-oxidobenzylidene) -1,2-diaminobenzene] (Costes *et al.*, 2000) in CH_3_CN (25 ml) was added dropwise to a solution of LiTCNQ (0.044 g, 0.2 mmol) in H_2_O (20 ml). The reaction was carried out under nitrogen atmosphere, using standard Schlenk techniques and degassed solvents. Reddish brown single crystals suitable for X-ray analysis were obtained in five days. Analysis, calculated for C_25_H_23_CoN_4_O_5_: C 57.81, H 4.66, N 10.79; found: C 57.76, H 4.74, N 10.83%.

### Refinement

H atoms bound to C atoms were positioned geometrically and refined as riding atoms, with C—H = 0.93 (aromatic) and 0.96 (methyl) Å and with *U*_iso_(H) = 1.2(1.5 for methyl)*U*_eq_(C). The water H atoms were initially located in a difference Fourier map and then treated as riding atoms, with O—H = 0.85 Å and *U*_iso_(H) = 1.5*U*_eq_(O).

### Crystal data

**Table:**

[Co(C_22_H_18_N_2_O_4_)(CN)(H_2_O)]·C_2_H_3_N	*F*(000) = 1072
*M**_r_* = 518.40	*D*_x_ = 1.446 Mg m^−^^3^
Monoclinic, *P*2_1_/*c*	Mo *K*α radiation, λ = 0.71073 Å
Hall symbol: -P 2ybc	Cell parameters from 17687 reflections
*a* = 10.829 (2) Å	θ = 3.1–27.5°
*b* = 13.209 (3) Å	µ = 0.77 mm^−^^1^
*c* = 18.906 (6) Å	*T* = 293 K
β = 118.30 (2)°	Block, brown
*V* = 2381.1 (10) Å^3^	0.34 × 0.31 × 0.29 mm
*Z* = 4	

### Data collection

**Table:**

Rigaku R-AXIS RAPID diffractometer	5426 independent reflections
Radiation source: fine-focus sealed tube	4439 reflections with *I* > 2σ(*I*)
graphite	*R*_int_ = 0.034
ω scans	θ_max_ = 27.5°, θ_min_ = 3.1°
Absorption correction: multi-scan (*ABSCOR*; Higashi, 1995)	*h* = −13→14
*T*_min_ = 0.780, *T*_max_ = 0.811	*k* = −17→17
22543 measured reflections	*l* = −24→24

### Refinement

**Table:**

Refinement on *F*^2^	Primary atom site location: structure-invariant direct methods
Least-squares matrix: full	Secondary atom site location: difference Fourier map
*R*[*F*^2^ > 2σ(*F*^2^)] = 0.034	Hydrogen site location: inferred from neighbouring sites
*wR*(*F*^2^) = 0.096	H-atom parameters constrained
*S* = 1.08	*w* = 1/[σ^2^(*F*_o_^2^) + (0.046*P*)^2^ + 0.9325*P*] where *P* = (*F*_o_^2^ + 2*F*_c_^2^)/3
5426 reflections	(Δ/σ)_max_ = 0.001
319 parameters	Δρ_max_ = 0.36 e Å^−^^3^
0 restraints	Δρ_min_ = −0.35 e Å^−^^3^

### Fractional atomic coordinates and isotropic or equivalent isotropic displacement parameters (Å^2^)

**Table:**

	*x*	*y*	*z*	*U*_iso_*/*U*_eq_	
C1	0.5547 (3)	0.1426 (2)	−0.19502 (14)	0.0647 (8)	
H1A	0.5301	0.0812	−0.2259	0.097*	
H1B	0.5808	0.1932	−0.2219	0.097*	
H1C	0.4757	0.1661	−0.1896	0.097*	
C2	0.7165 (2)	0.20216 (16)	−0.06373 (12)	0.0371 (4)	
C3	0.6721 (2)	0.30104 (17)	−0.08039 (14)	0.0469 (5)	
H3	0.6029	0.3184	−0.1317	0.056*	
C4	0.7297 (3)	0.37577 (17)	−0.02117 (15)	0.0504 (6)	
H4	0.6997	0.4425	−0.0334	0.060*	
C5	0.8296 (2)	0.35112 (16)	0.05426 (14)	0.0441 (5)	
H5	0.8671	0.4012	0.0934	0.053*	
C6	0.8773 (2)	0.24945 (14)	0.07391 (12)	0.0341 (4)	
C7	0.8218 (2)	0.17321 (14)	0.01489 (11)	0.0312 (4)	
C8	0.9842 (2)	0.22983 (15)	0.15339 (12)	0.0333 (4)	
H8	1.0159	0.2846	0.1885	0.040*	
C9	1.1540 (2)	0.13197 (15)	0.26124 (11)	0.0318 (4)	
C10	1.2143 (2)	0.21081 (16)	0.31618 (12)	0.0405 (5)	
H10	1.1825	0.2769	0.3018	0.049*	
C11	1.3210 (2)	0.18993 (18)	0.39164 (13)	0.0469 (5)	
H11	1.3610	0.2422	0.4286	0.056*	
C12	1.3696 (2)	0.09200 (19)	0.41328 (13)	0.0466 (5)	
H12	1.4418	0.0790	0.4647	0.056*	
C13	1.3121 (2)	0.01354 (17)	0.35951 (12)	0.0401 (5)	
H13	1.3456	−0.0521	0.3743	0.048*	
C14	1.2030 (2)	0.03317 (14)	0.28252 (11)	0.0316 (4)	
C15	1.1581 (2)	−0.13640 (15)	0.23320 (11)	0.0323 (4)	
H15	1.2264	−0.1561	0.2841	0.039*	
C16	0.9948 (2)	−0.19407 (14)	0.09435 (11)	0.0300 (4)	
C17	1.0922 (2)	−0.21447 (14)	0.17560 (11)	0.0324 (4)	
C18	1.1305 (2)	−0.31644 (15)	0.20116 (13)	0.0397 (5)	
H18	1.1953	−0.3296	0.2544	0.048*	
C19	1.0737 (3)	−0.39453 (16)	0.14897 (14)	0.0455 (5)	
H19	1.0989	−0.4608	0.1666	0.055*	
C20	0.9769 (2)	−0.37543 (15)	0.06817 (13)	0.0419 (5)	
H20	0.9385	−0.4292	0.0326	0.050*	
C21	0.9386 (2)	−0.27817 (15)	0.04140 (12)	0.0345 (4)	
C22	0.7825 (3)	−0.32967 (19)	−0.09336 (14)	0.0562 (7)	
H22A	0.7288	−0.3007	−0.1458	0.084*	
H22B	0.7218	−0.3684	−0.0797	0.084*	
H22C	0.8537	−0.3731	−0.0932	0.084*	
C23	0.8551 (2)	−0.00903 (15)	0.15346 (12)	0.0348 (4)	
Co1	0.99408 (3)	0.018833 (18)	0.124299 (14)	0.02757 (9)	
N1	1.04207 (17)	0.14260 (11)	0.18179 (9)	0.0293 (3)	
N2	1.13171 (17)	−0.03998 (12)	0.22136 (9)	0.0296 (3)	
N4	0.7711 (2)	−0.02985 (16)	0.17105 (14)	0.0538 (5)	
O1	0.95350 (15)	−0.10306 (10)	0.06457 (7)	0.0332 (3)	
O2	0.86109 (14)	0.07822 (10)	0.02591 (8)	0.0343 (3)	
O3	0.84670 (17)	−0.25085 (11)	−0.03599 (8)	0.0434 (4)	
O4	0.66969 (16)	0.12382 (12)	−0.11736 (9)	0.0465 (4)	
O5	1.14913 (14)	0.04801 (10)	0.09656 (8)	0.0339 (3)	
H51	1.1136	0.0809	0.0527	0.051*	
H52	1.1992	0.0032	0.0901	0.051*	
N3	1.4525 (3)	−0.2365 (3)	0.39165 (18)	0.1011 (11)	
C24	1.4969 (3)	−0.3138 (3)	0.41113 (18)	0.0685 (8)	
C25	1.5580 (4)	−0.4129 (3)	0.4384 (3)	0.1254 (18)	
H25A	1.6559	−0.4058	0.4759	0.188*	
H25B	1.5474	−0.4524	0.3933	0.188*	
H25C	1.5113	−0.4461	0.4643	0.188*	

### Atomic displacement parameters (Å^2^)

**Table:**

	*U*^11^	*U*^22^	*U*^33^	*U*^12^	*U*^13^	*U*^23^
C1	0.0577 (16)	0.0727 (19)	0.0373 (12)	0.0148 (13)	0.0009 (11)	0.0020 (12)
C2	0.0379 (11)	0.0340 (10)	0.0368 (10)	0.0067 (8)	0.0156 (9)	0.0031 (9)
C3	0.0467 (13)	0.0404 (12)	0.0450 (12)	0.0148 (10)	0.0148 (10)	0.0137 (10)
C4	0.0585 (14)	0.0275 (10)	0.0612 (14)	0.0144 (10)	0.0251 (12)	0.0115 (11)
C5	0.0518 (13)	0.0264 (10)	0.0522 (13)	0.0078 (9)	0.0231 (11)	0.0017 (10)
C6	0.0374 (10)	0.0254 (9)	0.0409 (10)	0.0058 (8)	0.0197 (9)	0.0021 (8)
C7	0.0333 (10)	0.0283 (9)	0.0353 (10)	0.0063 (7)	0.0191 (8)	0.0039 (8)
C8	0.0424 (11)	0.0246 (9)	0.0371 (10)	0.0006 (8)	0.0221 (9)	−0.0045 (8)
C9	0.0343 (10)	0.0296 (10)	0.0297 (9)	−0.0027 (8)	0.0137 (8)	−0.0032 (8)
C10	0.0450 (12)	0.0314 (10)	0.0392 (11)	−0.0020 (9)	0.0152 (9)	−0.0068 (9)
C11	0.0490 (13)	0.0429 (13)	0.0393 (11)	−0.0096 (10)	0.0132 (10)	−0.0138 (10)
C12	0.0419 (12)	0.0524 (14)	0.0317 (10)	−0.0052 (10)	0.0063 (9)	−0.0039 (10)
C13	0.0396 (11)	0.0374 (11)	0.0342 (10)	−0.0005 (9)	0.0101 (9)	0.0020 (9)
C14	0.0340 (10)	0.0298 (10)	0.0297 (9)	−0.0024 (7)	0.0142 (8)	−0.0015 (8)
C15	0.0366 (10)	0.0300 (10)	0.0286 (9)	0.0033 (8)	0.0142 (8)	0.0044 (8)
C16	0.0376 (10)	0.0213 (8)	0.0336 (9)	0.0009 (7)	0.0190 (8)	0.0002 (8)
C17	0.0391 (11)	0.0245 (9)	0.0338 (9)	0.0024 (8)	0.0176 (8)	0.0027 (8)
C18	0.0493 (12)	0.0290 (10)	0.0386 (11)	0.0070 (9)	0.0192 (10)	0.0072 (9)
C19	0.0622 (15)	0.0221 (10)	0.0511 (13)	0.0055 (9)	0.0261 (11)	0.0056 (9)
C20	0.0582 (14)	0.0241 (10)	0.0439 (11)	−0.0022 (9)	0.0246 (10)	−0.0056 (9)
C21	0.0416 (11)	0.0273 (10)	0.0340 (10)	−0.0020 (8)	0.0175 (9)	−0.0022 (8)
C22	0.0664 (16)	0.0424 (13)	0.0420 (12)	−0.0086 (12)	0.0110 (12)	−0.0140 (11)
C23	0.0384 (11)	0.0269 (9)	0.0352 (10)	−0.0009 (8)	0.0143 (9)	−0.0044 (8)
Co1	0.03420 (15)	0.02039 (13)	0.02460 (13)	0.00213 (10)	0.01105 (10)	−0.00039 (10)
N1	0.0368 (9)	0.0240 (8)	0.0277 (7)	0.0003 (6)	0.0158 (7)	−0.0023 (6)
N2	0.0343 (8)	0.0262 (8)	0.0264 (7)	0.0000 (6)	0.0130 (6)	−0.0009 (6)
N4	0.0546 (12)	0.0493 (12)	0.0678 (14)	−0.0096 (10)	0.0373 (11)	−0.0099 (10)
O1	0.0463 (8)	0.0212 (6)	0.0261 (6)	0.0023 (5)	0.0123 (6)	−0.0006 (5)
O2	0.0420 (8)	0.0260 (7)	0.0286 (6)	0.0069 (6)	0.0116 (6)	0.0004 (5)
O3	0.0547 (9)	0.0283 (7)	0.0341 (7)	−0.0033 (6)	0.0104 (7)	−0.0058 (6)
O4	0.0471 (9)	0.0422 (9)	0.0346 (8)	0.0103 (7)	0.0067 (7)	0.0018 (7)
O5	0.0400 (7)	0.0303 (7)	0.0324 (7)	0.0050 (6)	0.0180 (6)	0.0024 (6)
N3	0.087 (2)	0.088 (2)	0.088 (2)	0.0309 (18)	0.0079 (17)	0.0136 (18)
C24	0.0522 (16)	0.073 (2)	0.0594 (16)	0.0092 (14)	0.0092 (13)	−0.0069 (15)
C25	0.083 (3)	0.059 (2)	0.185 (5)	0.0098 (19)	0.023 (3)	−0.001 (3)

### Geometric parameters (Å, °)

**Table:**

C1—O4	1.426 (3)	C15—C17	1.422 (3)
C1—H1A	0.9600	C15—H15	0.9300
C1—H1B	0.9600	C16—O1	1.313 (2)
C1—H1C	0.9600	C16—C17	1.417 (3)
C2—O4	1.367 (3)	C16—C21	1.424 (3)
C2—C3	1.376 (3)	C17—C18	1.425 (3)
C2—C7	1.431 (3)	C18—C19	1.357 (3)
C3—C4	1.398 (3)	C18—H18	0.9300
C3—H3	0.9300	C19—C20	1.406 (3)
C4—C5	1.359 (3)	C19—H19	0.9300
C4—H4	0.9300	C20—C21	1.371 (3)
C5—C6	1.424 (3)	C20—H20	0.9300
C5—H5	0.9300	C21—O3	1.372 (2)
C6—C7	1.409 (3)	C22—O3	1.424 (3)
C6—C8	1.420 (3)	C22—H22A	0.9600
C7—O2	1.309 (2)	C22—H22B	0.9600
C8—N1	1.300 (2)	C22—H22C	0.9600
C8—H8	0.9300	C23—N4	1.140 (3)
C9—C14	1.394 (3)	Co1—C23	1.869 (2)
C9—C10	1.395 (3)	Co1—N1	1.8944 (15)
C9—N1	1.421 (2)	Co1—N2	1.8972 (16)
C10—C11	1.372 (3)	Co1—O1	1.8948 (13)
C10—H10	0.9300	Co1—O2	1.8998 (14)
C11—C12	1.383 (3)	Co1—O5	2.0194 (14)
C11—H11	0.9300	O5—H51	0.8500
C12—C13	1.377 (3)	O5—H52	0.8500
C12—H12	0.9300	N3—C24	1.114 (4)
C13—C14	1.397 (3)	C24—C25	1.447 (5)
C13—H13	0.9300	C25—H25A	0.9600
C14—N2	1.421 (2)	C25—H25B	0.9600
C15—N2	1.301 (2)	C25—H25C	0.9600
			
O4—C1—H1A	109.5	C19—C18—C17	120.90 (19)
O4—C1—H1B	109.5	C19—C18—H18	119.6
H1A—C1—H1B	109.5	C17—C18—H18	119.6
O4—C1—H1C	109.5	C18—C19—C20	120.06 (19)
H1A—C1—H1C	109.5	C18—C19—H19	120.0
H1B—C1—H1C	109.5	C20—C19—H19	120.0
O4—C2—C3	125.62 (19)	C21—C20—C19	120.55 (19)
O4—C2—C7	113.63 (17)	C21—C20—H20	119.7
C3—C2—C7	120.7 (2)	C19—C20—H20	119.7
C2—C3—C4	120.8 (2)	C20—C21—O3	125.44 (18)
C2—C3—H3	119.6	C20—C21—C16	121.19 (19)
C4—C3—H3	119.6	O3—C21—C16	113.37 (17)
C5—C4—C3	120.1 (2)	O3—C22—H22A	109.5
C5—C4—H4	119.9	O3—C22—H22B	109.5
C3—C4—H4	119.9	H22A—C22—H22B	109.5
C4—C5—C6	120.6 (2)	O3—C22—H22C	109.5
C4—C5—H5	119.7	H22A—C22—H22C	109.5
C6—C5—H5	119.7	H22B—C22—H22C	109.5
C7—C6—C8	122.36 (17)	N4—C23—Co1	177.41 (19)
C7—C6—C5	120.13 (19)	C23—Co1—N1	92.35 (8)
C8—C6—C5	117.48 (19)	C23—Co1—O1	90.96 (7)
O2—C7—C6	125.07 (17)	N1—Co1—O1	176.68 (7)
O2—C7—C2	117.38 (17)	C23—Co1—N2	90.42 (8)
C6—C7—C2	117.53 (17)	N1—Co1—N2	85.55 (7)
N1—C8—C6	126.20 (18)	O1—Co1—N2	94.73 (6)
N1—C8—H8	116.9	C23—Co1—O2	91.57 (8)
C6—C8—H8	116.9	N1—Co1—O2	94.58 (6)
C14—C9—C10	120.28 (18)	O1—Co1—O2	85.03 (6)
C14—C9—N1	114.50 (16)	N2—Co1—O2	178.00 (7)
C10—C9—N1	125.23 (18)	C23—Co1—O5	178.11 (7)
C11—C10—C9	119.3 (2)	N1—Co1—O5	87.05 (6)
C11—C10—H10	120.3	O1—Co1—O5	89.65 (6)
C9—C10—H10	120.3	N2—Co1—O5	87.75 (6)
C10—C11—C12	120.7 (2)	O2—Co1—O5	90.26 (6)
C10—C11—H11	119.7	C8—N1—C9	121.80 (16)
C12—C11—H11	119.7	C8—N1—Co1	125.49 (14)
C13—C12—C11	120.7 (2)	C9—N1—Co1	112.69 (12)
C13—C12—H12	119.7	C15—N2—C14	122.36 (16)
C11—C12—H12	119.7	C15—N2—Co1	125.09 (13)
C12—C13—C14	119.5 (2)	C14—N2—Co1	112.53 (12)
C12—C13—H13	120.2	C16—O1—Co1	125.94 (12)
C14—C13—H13	120.2	C7—O2—Co1	126.13 (12)
C9—C14—C13	119.50 (18)	C21—O3—C22	117.74 (17)
C9—C14—N2	114.59 (16)	C2—O4—C1	117.87 (19)
C13—C14—N2	125.90 (18)	Co1—O5—H51	107.7
N2—C15—C17	126.06 (18)	Co1—O5—H52	124.8
N2—C15—H15	117.0	H51—O5—H52	103.9
C17—C15—H15	117.0	N3—C24—C25	178.4 (4)
O1—C16—C17	124.47 (17)	C24—C25—H25A	109.5
O1—C16—C21	117.89 (17)	C24—C25—H25B	109.5
C17—C16—C21	117.64 (17)	H25A—C25—H25B	109.5
C16—C17—C15	122.53 (17)	C24—C25—H25C	109.5
C16—C17—C18	119.65 (18)	H25A—C25—H25C	109.5
C15—C17—C18	117.79 (18)	H25B—C25—H25C	109.5

### Hydrogen-bond geometry (Å, °)

**Table:**

*D*—H···*A*	*D*—H	H···*A*	*D*···*A*	*D*—H···*A*
O5—H51···O3^i^	0.85	2.33	2.922 (2)	127
O5—H51···O1^i^	0.85	2.00	2.799 (2)	156
O5—H52···O2^i^	0.85	2.24	2.813 (2)	124
O5—H52···O4^i^	0.85	2.10	2.902 (2)	158
C10—H10···N4^ii^	0.93	2.61	3.433 (3)	148
C15—H15···N3	0.93	2.56	3.440 (3)	159

Symmetry codes: (i) −*x*+2, −*y*, −*z*; (ii) −*x*+2, *y*+1/2, −*z*+1/2.
